# Smartphone Screen Time, Sleep Quality, and Headache in Young Adults Presenting to the Emergency Department: A Case–Control Study

**DOI:** 10.3390/healthcare14162657

**Published:** 2026-08-21

**Authors:** Yusuf Kantar, Yusuf Ertuğrul Aslan, Arif Onur Eden, Yasin Bilgin, Orhan Tanrıverdi, Abdullah Emre Yurttutan, Erdem Yakup Çimen, Fatih Mehmet Sarı

**Affiliations:** 1 Department of Emergency Medicine, Faculty of Medicine, Erzincan Binali Yıldırım University, Erzincan 24100, Türkiye; 2 Department of Emergency Medicine, Faculty of Medicine, Erciyes University, Kayseri 38039, Türkiye

**Keywords:** smartphone screen time, sleep quality, headache, young adults, emergency department

## Abstract

**Background**: Smartphone screen exposure has increased substantially among young adults and has been associated with headache and sleep disturbances. This study aimed to investigate the associations between smartphone screen time, headache severity, sleep duration, and sleep quality in young adults presenting to the emergency department with headache. **Methods**: This single-center case–control study was conducted in the emergency department of a tertiary university hospital between November 2025 and April 2026. A total of 300 participants aged 18–40 years were enrolled, including 150 patients with headache and 150 controls without headache. Daily smartphone screen time was obtained from the built-in smartphone records for the preceding completed seven-day period, headache severity was assessed using the 11-point Numeric Rating Scale, and sleep quality was evaluated using the validated Turkish version of the Single-Item Sleep Quality Scale. **Results**: Participants with headache had longer smartphone screen time (7.0 vs. 5.0 h/day; *p* < 0.001), shorter sleep duration (7.0 vs. 8.0 h/day; *p* = 0.005), and poorer sleep quality (5.0 vs. 7.0; *p* < 0.001) than controls. Screen time correlated with headache severity (ρ = 0.430, *p* < 0.001) and inversely with sleep quality after adjustment for study group (partial ρ = −0.185, *p* = 0.001). Nonlinear multivariable modeling showed an association with headache presentation (*p* < 0.001); relative to 6 h/day, the aOR at 7 h/day was 8.83 (95% CI: 5.00–15.60), with a plateau at higher exposures. **Conclusions**: Longer device-recorded smartphone screen time was associated with headache presentation and severity in young adults. Participants with headache also reported poorer sleep quality and shorter sleep duration. These findings suggest relevant associations between smartphone exposure, sleep, and headache but do not establish causality or a clinical screen-time threshold. Prospective studies are warranted.

## 1. Background

### 1.1. Smartphone/Screen Exposure

The use of screen-based devices, including televisions, mobile phones, tablets, and computers, has become increasingly common among young adults [1]. Although the continuous increase in screen time among adolescents and young adults has become an inevitable consequence of the digital age, it is also accompanied by a range of adverse outcomes. Beyond screen exposure duration itself, problematic or addictive patterns of screen use have also been associated with a wide range of psychological, cognitive, and physical health problems [2]. Individuals who grew up with digital screens—including gaming consoles, televisions, and computers—now constitute today’s young adult population, and despite changes in device types, overall screen exposure continues to increase [3]. Recent evidence from Türkiye indicates that prolonged smartphone use is common among young adults. Studies conducted in young adult and university student populations have reported that approximately 70–77% of participants use smartphones for more than 4 h per day [4]. In addition, smartphone use has been examined in relation to sleep quality among Turkish university students [5]. These findings underscore the substantial smartphone exposure among young adults in Türkiye and highlight the need to further investigate its potential relationship with sleep and headache in emergency department (ED) populations. During the COVID-19 pandemic, screen time among young adults increased substantially as social, educational, and occupational activities increasingly shifted to digital platforms [6]. Although the pandemic period has passed, increased screen exposure has persisted among young adults. Studies conducted particularly among university students have shown that increased screen exposure is associated with poorer mental health, a higher prevalence of headaches, and sleep problems [1,7]. Among various screen-based devices, smartphones have become an important source of daily screen exposure among young adults because of their continuous accessibility and integration into academic, occupational, and social activities. In the present study, screen exposure refers specifically to smartphone screen time, rather than broader patterns of smartphone use or problematic/addictive use. Furthermore, built-in smartphone screen-time tracking systems provide readily accessible information on recent smartphone use and may reduce reliance on self-reported estimates of screen time.

### 1.2. Sleep Duration and Sleep Quality

Sleep duration and sleep quality are well-established determinants of overall health. Global studies have shown that the majority of young adults worldwide sleep less than eight hours per day. Prolonged daily screen time, particularly when combined with bedtime use of digital devices, may further increase the likelihood of adverse health outcomes [8]. Among these adverse outcomes, headache is one of the most frequently reported complaints. Numerous studies have shown that as sleep quality deteriorates and sleep duration decreases, the frequency and severity of headaches increase [9].

### 1.3. Headache and Possible Mechanisms

Growing evidence suggests that the relationship between smartphone use, sleep disturbances, visual strain and headache may involve interconnected behavioral and biological mechanisms. Prolonged smartphone use, particularly during evening hours, may interfere with normal sleep patterns through behavioral displacement of sleep and exposure to screen-emitted light, which may affect circadian timing and sleep onset. In addition, prolonged near-screen viewing may increase visual demand and contribute to ocular discomfort and visual strain, which may trigger or aggravate headache symptoms. Smartphone use may also involve sustained forward-head and neck postures, potentially increasing cervical musculoskeletal tension and contributing to headache. Sleep deprivation and poor sleep quality may further lower the threshold for headache occurrence and exacerbate headache severity [2]. Taken together, these mechanisms provide a plausible framework linking smartphone screen time with sleep disturbances and headache, although the observational design of the present study does not allow causal inferences.

### 1.4. Emergency Department Context and the Research Gap

Most previous studies have evaluated these associations in community-based populations, university students, or patients attending outpatient clinics. These populations may differ from ED patients in terms of acute symptom burden, reasons for healthcare presentation, and the clinical context in which headache is evaluated, which may limit the direct generalizability of their findings to emergency department populations. In contrast, evidence regarding the relationship between smartphone screen time, sleep characteristics, and headache among young adults presenting to the ED remains limited. The ED therefore provides a distinct and clinically relevant setting in which these associations can be examined among patients seeking medical care for an acute headache complaint.

### 1.5. Study Aim

Therefore, this study aimed to investigate the associations between smartphone screen time, headache severity, sleep duration, and sleep quality in young adults presenting to the ED with headache, compared with controls without headache within the same age range.

## 2. Methods

### 2.1. Study Design and Setting

This prospective, single-center, case–control study was conducted in the ED of Erzincan Binali Yıldırım University Hospital, Türkiye, between November 2025 and April 2026. The study hospital is a tertiary referral center serving the city as well as the surrounding districts and neighboring provinces, with approximately 1500–2000 ED visits per day and a broad range of emergency presentations.

The study was conducted in accordance with the ethical principles of the Declaration of Helsinki and was approved by the Erzincan Binali Yıldırım University Faculty of Medicine Ethics Committee (Approval No. 2025-18/08; 16 October 2025). Written informed consent was obtained from all participants before their enrollment in the study. The study was reported in accordance with the Strengthening the Reporting of Observational Studies in Epidemiology (STROBE) recommendations specifically developed for the reporting of case–control studies.

### 2.2. Participants and Study Groups

Participants were recruited consecutively throughout the study period according to predefined eligibility criteria. Case and control participants were recruited concurrently rather than during separate recruitment periods, with no restriction according to day of the week or work shift. The same age eligibility range was applied to both study groups; no individual or frequency matching by age was performed.

Young adults aged 18–40 years who presented to the ED on an outpatient basis were included in the study. All participants were given detailed verbal information prior to the study, and those who agreed to participate were asked to sign an informed consent declaration form. Both case and control participants were required to be classified as green triage according to the triage system defined by the Turkish Ministry of Health [10]. All patients presenting with headache underwent a standardized initial clinical assessment, including medical history, vital sign measurement, physical examination, and neurological examination. Headache subtypes were not prospectively classified according to the International Classification of Headache Disorders or other formal diagnostic criteria. Accordingly, the headache group represented a clinically heterogeneous population of young adults presenting to the ED with headache rather than a cohort with a specific primary headache disorder. Additional diagnostic investigations, including laboratory tests and imaging, were performed when clinically indicated rather than routinely in all patients. Patients in whom the clinical assessment or additional investigations identified an underlying condition that could account for the headache were excluded from the case group.

The study population consisted of two groups: a case group and a control group, each comprising 150 participants. The case group included patients aged 18–40 years who presented to the ED with complaints of headache. The control group consisted of non-trauma patients aged 18–40 years who met the green-triage criteria defined by the Turkish Ministry of Health [10], indicating a stable, non-urgent clinical condition suitable for outpatient management. Controls presented with complaints other than headache and reported no headache at the time of enrollment. No individual or frequency matching was performed between cases and controls. Rather than recruiting healthy community volunteers, the control group was selected from patients presenting to the same ED to ensure that both groups were evaluated under comparable conditions within the same clinical setting.

General exclusion criteria were age < 18 or >40 years, refusal to participate, inability to provide informed consent because of impaired consciousness, red-triage/high-acuity status, traumatic presentation, inability to provide the required smartphone screen-time record, or absence or disabled status of the relevant screen-time tracking function on the participant’s smartphone. Patients with a secondary cause of headache identified during ED evaluation were additionally excluded from the headache group.

**Figure 1:**
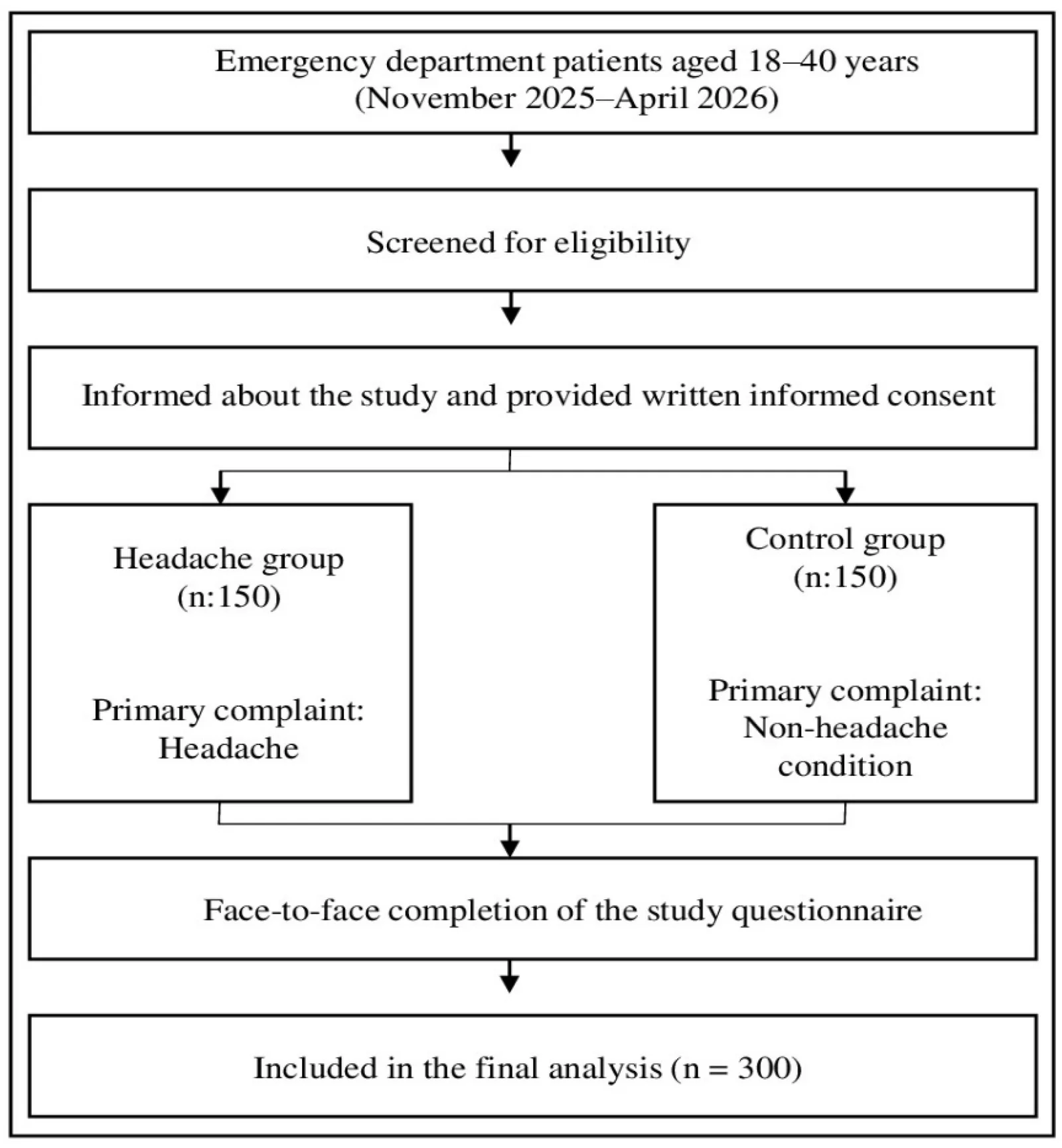
Participant flow diagram.

A total of 300 participants were included in the final analytical sample, comprising 150 participants in the headache group and 150 in the control group. Participant recruitment and study-group allocation are summarized in Figure 1.

### 2.3. Data Collection and Measurements

Data were collected using a structured data collection form developed by the authors based on a review of the relevant literature and the consensus of five emergency medicine specialists, each with at least five years of professional experience. The form was designed to facilitate standardized and time-efficient data collection in the ED setting. Before study recruitment began, it was pilot-tested in patients with characteristics similar to those of the target population to assess its clarity and feasibility. No formal psychometric validation or reproducibility testing was performed for the author-developed components of the data collection form. Participants completed the data collection form under the supervision of the researchers during face-to-face interviews. First, the participants’ age and sex were documented. Participants in both groups were asked whether they had a previously established diagnosis of migraine. Migraine history was recorded as a pre-existing clinical characteristic and was not used to classify the current headache presentation. Headache presentations in the case group were not further classified into specific headache disorders according to formal diagnostic criteria. No personally identifying information was recorded on the study form. The data collection form is provided as Supplementary Materials.

#### 2.3.1. Headache Characteristics

Patients in the case group were additionally asked to report their usual headache frequency using three predefined categories: less than once per week, once per week, or more than once per week. This simple categorical assessment was intended to provide a descriptive characterization of headache frequency within the case group rather than a comprehensive measure of headache burden.

Headache severity was assessed using an 11-point Numeric Rating Scale (NRS; 0 = no pain, 10 = worst imaginable pain), a validated measure of acute pain intensity in adult ED patients accompanied by pain descriptors [11]. This section of the data collection form was left blank for participants in the control group.

Because headache phenotypes were not prospectively classified according to standardized diagnostic criteria, no distinction among migraine, tension-type headache, or other primary headache subtypes was made in the study analyses.

#### 2.3.2. Smartphone Screen Time

**Figure 2:**
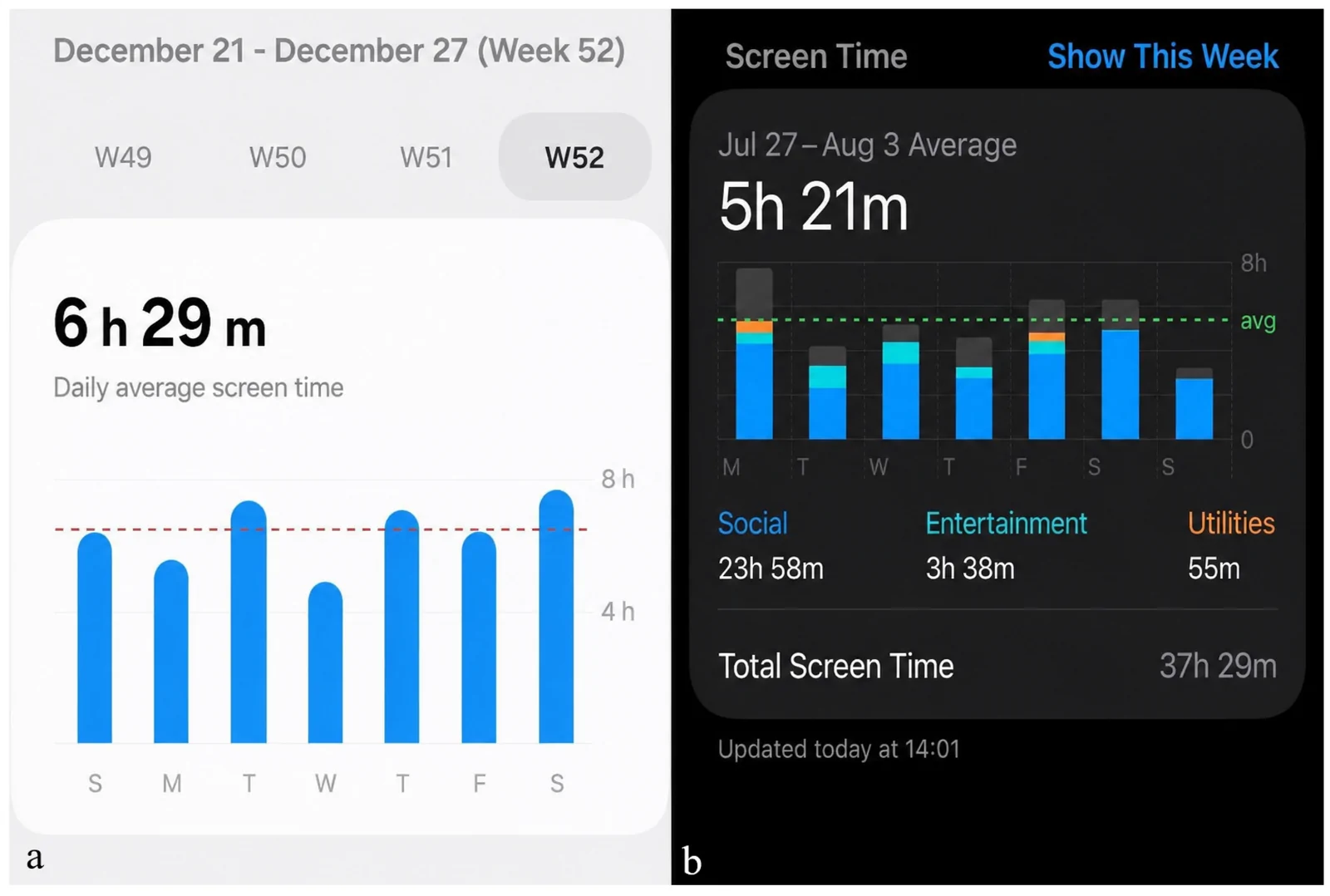
Examples of the built-in smartphone screen-time displays used to obtain average daily screen time: (**a**) Android; (**b**) iOS.

Smartphone screen time was obtained directly from the built-in screen-time tracking function available in each participant’s smartphone settings (Figure 2a,b) [12,13]. Participants using either iOS or Android devices were asked to open the weekly screen-time report on their smartphones, and the average daily screen time displayed for the preceding completed 7-day period was recorded. The day of ED presentation was not included because it represented an incomplete day of smartphone use. The same measurement procedure was applied to both operating systems, although the built-in tracking function provided by each device was used rather than a separate standardized monitoring application. Screen-time values were recorded in whole hours; values with <30 additional minutes were rounded down, whereas values with ≥30 additional minutes were rounded up to the nearest whole hour. This device-recorded approach was used to reduce reliance on participants’ recall of their smartphone use. The assessment was restricted to screen time recorded on the participant’s primary smartphone. Screen exposure from other devices (e.g., computers, tablets, and televisions), use of additional smartphones, application-specific use, and the purpose of smartphone use (e.g., occupational or non-occupational) were not assessed.

#### 2.3.3. Assessment of Sleep Duration and Sleep Quality

Participants were then asked to report their usual average daily sleep duration in hours by answering the question, ‘On average, how many hours do you usually sleep per day?’ No specific recall period was defined for this assessment. Finally, sleep quality over the previous 7 days was assessed using the validated Turkish version of the single-item Sleep Quality Scale (SQS), in which participants rated their overall sleep quality on an 11-point scale ranging from 0 to 10 [14,15]. In the original psychometric evaluation, the SQS demonstrated strong concurrent validity with established sleep-quality measures, with correlations of −0.76 with the Morning Questionnaire–Insomnia and −0.92 with the Pittsburgh Sleep Quality Index, while test–retest reliability coefficients ranged from 0.62 to 0.74 [12]. In the Turkish validation study, test–retest reliability was ICC = 0.82 (95% CI: 0.66–0.91), supporting the reliability and validity of the Turkish version [15].

### 2.4. Sample Size

An a priori sample size calculation was performed using G*Power version 3.1.9.7. The calculation was based on comparison of smartphone screen time between two independent groups. Assuming a moderate standardized difference (Cohen’s *d* = 0.50), a two-sided α level of 0.05, and 80% statistical power, the minimum required sample size was calculated as 64 participants per group (128 participants in total). The final analytical sample comprised 300 participants, with 150 participants in each study group.

### 2.5. Statistical Analysis

Statistical analyses were performed using IBM SPSS Statistics version 27.0 (IBM Corp., Armonk, NY, USA) and R software 4.5.2 (R Foundation for Statistical Computing, Vienna, Austria). Distributional characteristics of continuous variables were evaluated using graphical methods and the Shapiro–Wilk test. Because the principal continuous variables showed non-normal and discrete distributions, continuous variables are presented as median and interquartile range (IQR), whereas categorical variables are presented as number and percentage.

Continuous variables were compared between the headache and control groups using the Mann–Whitney *U* test. Categorical variables were compared using Pearson’s chi-square test. In addition to *p*-values, effect sizes were reported for between-group comparisons. Rank-biserial correlation was used as the effect-size measure for Mann–Whitney *U* comparisons, and Cramér’s *V* was used for categorical comparisons.

Associations among headache severity, smartphone screen time, sleep duration, and sleep quality were evaluated using Spearman’s rank correlation coefficient (ρ). Analyses involving headache severity were restricted to the headache group because headache severity was not measured in controls. Within the headache group, correlations were examined between headache severity and smartphone screen time, sleep duration, and sleep quality, as well as among smartphone screen time, sleep duration, and sleep quality.

The association between smartphone screen time and sleep quality was additionally examined separately in the headache and control groups. Because a correlation calculated across the entire sample could be influenced by systematic differences between the two study groups, the pooled association was further evaluated using partial Spearman correlation controlling for study-group membership. Evidence for a difference in the screen-time–sleep-quality association between groups was evaluated using a rank-based interaction model. In addition, the difference between the two group-specific Spearman correlation coefficients was assessed using 10,000 within-group bootstrap resamples and a percentile-based 95% confidence interval (CI).

For graphical presentation of correlation analyses, individual observations were displayed with jittering to reduce overplotting caused by the discrete nature of the variables. Smoothed curves and corresponding 95% confidence bands were generated using generalized additive models with shrinkage cubic regression splines. These smooths were included for visualization only; statistical inference regarding correlations was based on Spearman’s ρ and its corresponding *p*-value.

#### Multivariable Logistic Regression

The binary outcome for regression analyses was headache presentation, defined as membership in the headache group versus the control group. Multivariable logistic regression was used to estimate adjusted associations between smartphone screen time and headache presentation. Age, sex, and history of migraine were retained as prespecified adjustment variables rather than selected using an automated stepwise procedure.

Before interpretation of smartphone screen time as a continuous exposure, its functional relationship with the log odds of headache presentation was evaluated. A conventional linear specification was compared with nonlinear specifications using natural cubic splines. A natural cubic spline with 2 degrees of freedom provided a substantially better fit than the corresponding model assuming a constant linear effect. A more flexible 3-degree-of-freedom spline was also examined but produced unstable fitted probabilities in sparsely represented exposure regions. Therefore, the 2-degree-of-freedom natural cubic spline was retained as the primary nonlinear specification to balance model flexibility and parsimony.

The primary model included smartphone screen time modeled as a natural cubic spline with 2 degrees of freedom and adjustment for age, sex, and history of migraine. Because sleep duration and sleep quality may be related to pathways linking smartphone exposure with headache and their inclusion could therefore alter the interpretation of the exposure coefficient, these variables were not included in the primary adjustment set. An extended model additionally included sleep duration and sleep quality as a sensitivity analysis.

Overall evidence for an association between smartphone screen time and headache presentation was assessed using a likelihood-ratio test comparing the model containing the spline terms with the corresponding model without smartphone screen time. Evidence of nonlinearity was evaluated by comparing the spline model with the corresponding model in which smartphone screen time was entered as a single linear term.

Adjusted odds ratios (aORs) and 95% confidence intervals (CIs) derived from the spline model were estimated at 7, 8, and 9 h/day relative to 6 h/day. Six hours/day was selected as the reference because it was the lowest smartphone screen-time value observed in both the headache and control groups. Although regression models were fitted using the full analytical sample, graphical presentation of the nonlinear association was restricted to the common-support range of 6–9 h/day, within which observations from both study groups were available.

Multicollinearity among covariates was evaluated using variance inflation factors (VIFs). VIF values ranged from approximately 1.05 to 1.44, providing no evidence of problematic multicollinearity. Therefore, no variables were excluded on the basis of multicollinearity. The previously considered interpretation of a statistical suppressor effect was not used in the revised analysis.

There were no missing observations for the variables included in the principal analyses. All statistical tests were two-sided, and a *p*-value < 0.05 was considered statistically significant. Adjusted associations from logistic regression models are reported as aORs with 95% CIs.

## 3. Results

### 3.1. Participant Characteristics

A total of 300 participants were included in the analysis, comprising 150 participants presenting with headache and 150 ED controls without headache. There were no statistically significant between-group differences in age, sex distribution, or previous migraine diagnosis. Median age was 27.0 years (IQR, 21.0–33.0) in the headache group and 27.5 years (IQR, 22.0–34.0) in the control group (*p* = 0.366; rank-biserial correlation [*r_rb_*] = −0.060). Women accounted for 61.3% of the headache group and 53.3% of the control group (*p* = 0.161; Cramér’s *V* = 0.081), while a previous migraine diagnosis was reported by 22.7% and 18.7% of participants, respectively (*p* = 0.392; Cramér’s *V* = 0.049).

Device-recorded daily smartphone screen time was higher in the headache group than in the control group, with median values of 7.0 h/day (IQR, 7.0–8.0) and 5.0 h/day (IQR, 4.0–5.0), respectively (*p* < 0.001). The corresponding effect size was large (*r_rb_* = 0.857). Participants in the headache group also reported shorter usual daily sleep duration than controls (7.0 [IQR, 7.0–8.0] vs. 8.0 [IQR, 7.0–8.0] h/day; *p* = 0.005; *r_rb_* = −0.175) and lower sleep quality scores (5.0 [IQR, 4.0–6.0] vs. 7.0 [IQR, 6.0–8.0]; *p* < 0.001; rrb = −0.387) (Table 1).

### 3.2. Headache Characteristics and Correlation Analyses

Among participants in the headache group, 132 (88.0%) reported experiencing headache less than once per week, 11 (7.3%) reported headache once per week, and 7 (4.7%) reported headache more than once per week. Median headache severity at ED presentation was 6.0 (IQR, 4.0–7.0) on the NRS (Table 2).

**Figure 3:**
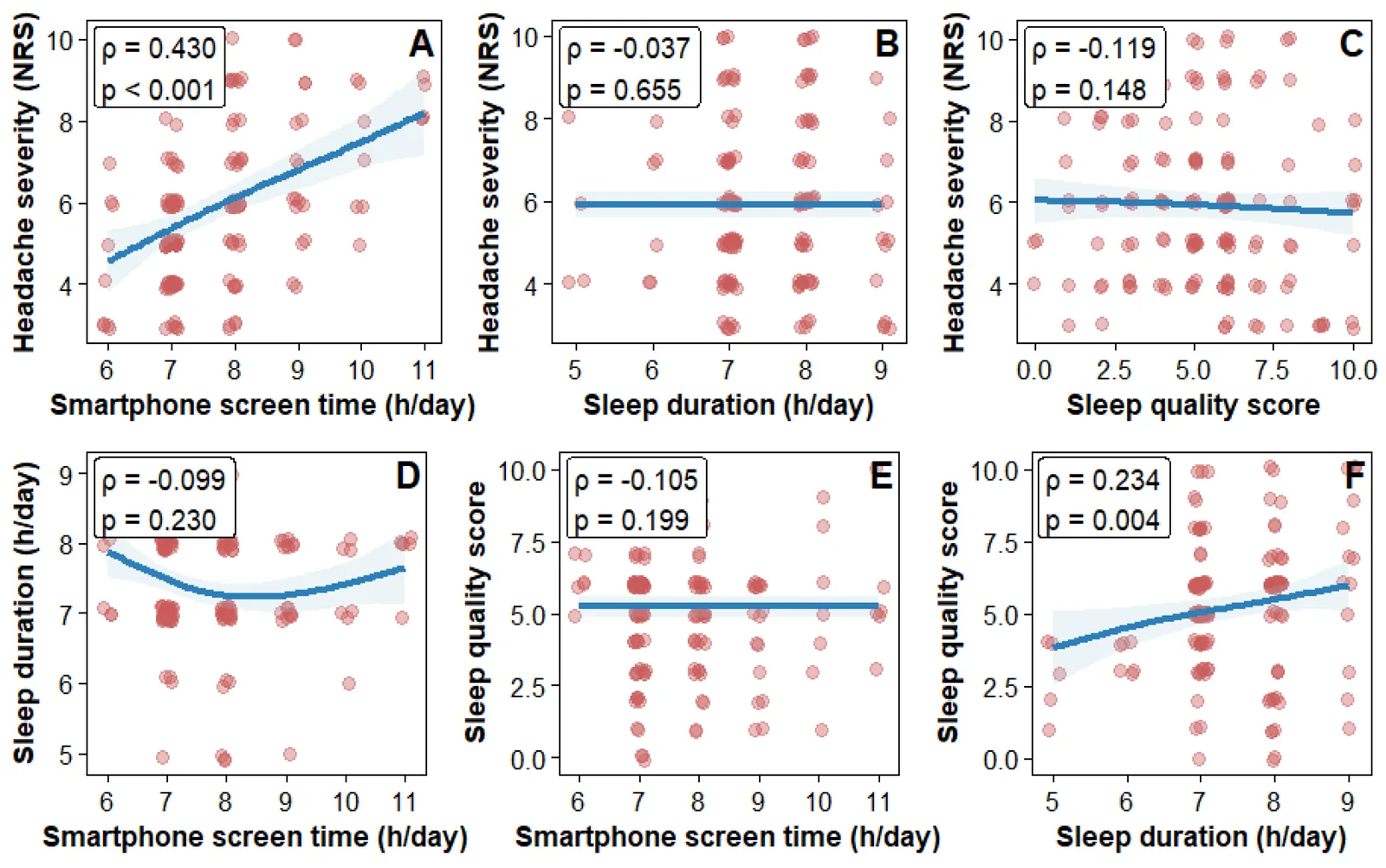
Correlations among headache severity, smartphone screen time, sleep duration, and sleep quality in the headache group. (**A**) Smartphone screen time and headache severity; (**B**) sleep duration and headache severity; (**C**) sleep quality and headache severity; (**D**) smartphone screen time and sleep duration; (**E**) smartphone screen time and sleep quality; and (**F**) sleep duration and sleep quality. Spearman’s rank correlation coefficients (ρ) and corresponding *p*-values are shown in each panel. Smoothed curves and shaded 95% confidence bands are provided for visualization only. Colored dots represent individual observations, solid curves represent smoothed trends, and shaded areas indicate 95% confidence bands. The smoothed curves are provided for visualization only; statistical inference is based on Spearman’s rank correlation coefficient (ρ).

Within the headache group, daily smartphone screen time showed a moderate positive correlation with headache severity (ρ = 0.430, *p* < 0.001). In contrast, headache severity was not significantly correlated with usual sleep duration (ρ = −0.037, *p* = 0.655) or sleep quality (ρ = −0.119, *p* = 0.148). Smartphone screen time was not significantly correlated with sleep duration (ρ = −0.099, *p* = 0.230) or sleep quality (ρ = −0.105, *p* = 0.199). A weak positive correlation was observed between sleep duration and sleep quality (ρ = 0.234, *p* = 0.004) (Figure 3).

### 3.3. Association Between Smartphone Screen Time and Sleep Quality

When all participants were analyzed together, daily smartphone screen time was inversely correlated with sleep quality (ρ = −0.370, *p* < 0.001). Because both smartphone screen time and sleep quality differed substantially between study groups, the pooled correlation was additionally examined after accounting for group membership. The partial Spearman correlation controlling for study group was weaker but remained statistically significant (partial ρ = −0.185, *p* = 0.001).

**Figure 4:**
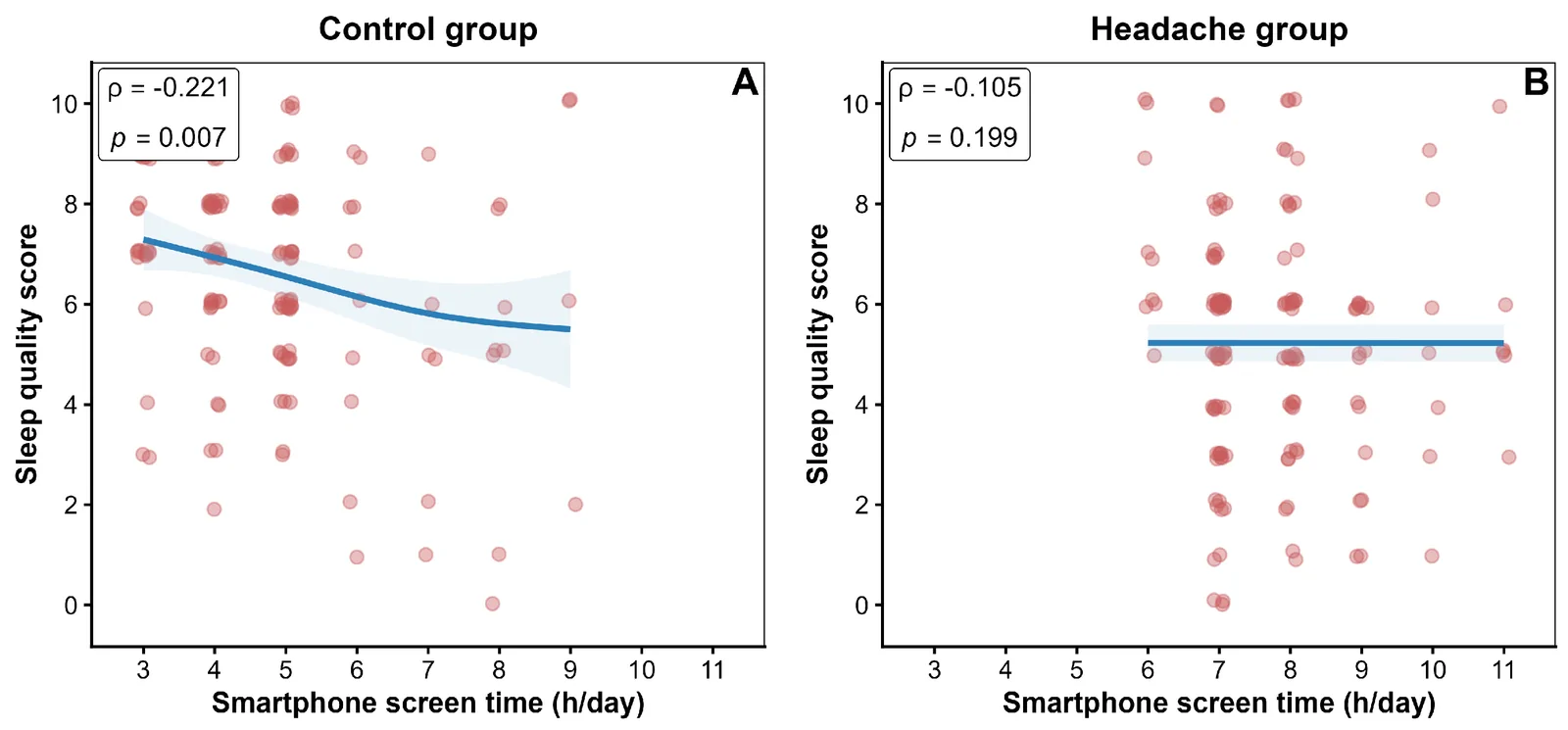
Associations between smartphone screen time and sleep quality according to study group. (**A**) Control group and (**B**) headache group. Spearman’s rank correlation coefficients (ρ) and corresponding *p*-values are shown in each panel. Smoothed curves and shaded 95% confidence bands are provided for visualization only.

In group-specific analyses, smartphone screen time showed a weak inverse correlation with sleep quality in the control group (ρ = −0.221, *p* = 0.007), whereas the corresponding correlation in the headache group was smaller and not statistically significant (ρ = −0.105, *p* = 0.199) (Figure 4).

Although statistical significance differed between the two subgroup analyses, there was no evidence that the magnitude of the association differed between groups. The screen-time-by-group interaction in the rank-based model was not statistically significant (*p* = 0.389). Consistently, the observed difference between the group-specific Spearman coefficients was Δρ = 0.116, with a bootstrap 95% CI ranging from −0.120 to 0.353, which included zero.

### 3.4. Multivariable Association Between Smartphone Screen Time and Headache Presentation

The association between smartphone screen time and headache presentation was nonlinear. Modeling screen time using a natural cubic spline with 2 degrees of freedom provided significantly better fit than modeling screen time as a single linear term (*p* < 0.001 for the nonlinear component). The overall association between smartphone screen time and headache presentation was also statistically significant (*p* < 0.001).

**Figure 5:**
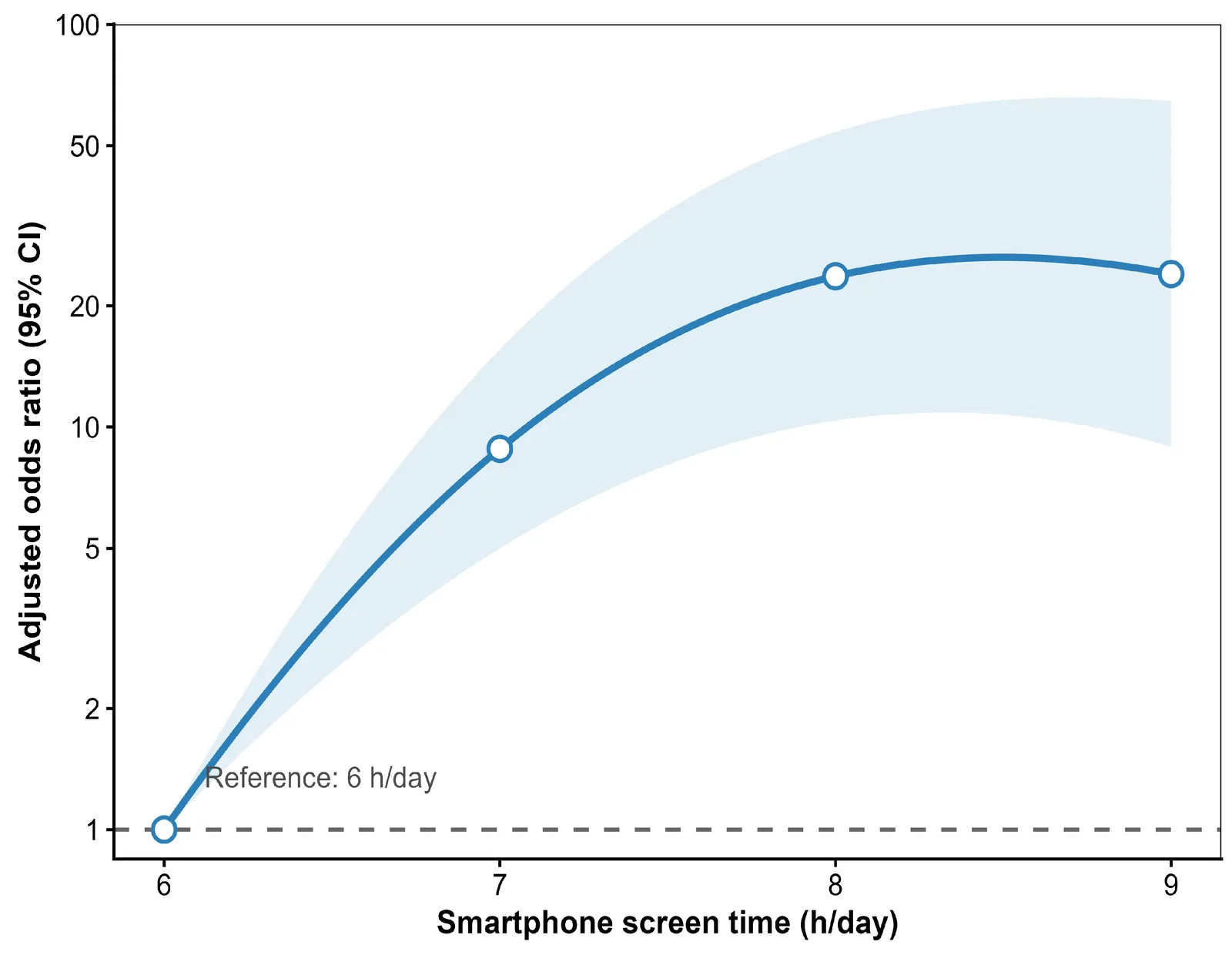
Nonlinear association between daily smartphone screen time and headache presentation. Adjusted odds ratios were estimated using multivariable logistic regression with smartphone screen time modeled as a natural cubic spline with 2 degrees of freedom and adjustment for age, sex, and previous migraine diagnosis. Six hours/day was used as the reference because it was the lowest screen-time value observed in both study groups. The shaded area represents the 95% confidence interval. The curve is displayed over the common-support range of 6–9 h/day, although the model was fitted using the full analytical sample (*n* = 300). The overall association and nonlinear component were both statistically significant (*p* < 0.001).

In the primary multivariable model adjusted for age, sex, and previous migraine diagnosis, using 6 h/day as the reference, the adjusted odds of headache presentation increased markedly across the common-support range. In the present study, screen exposure refers specifically to smartphone screen time, rather than broader patterns of smartphone use or problematic/addictive use. The aOR was 8.83 (95% CI, 5.00–15.60) at 7 h/day, 23.70 (95% CI, 10.38–54.10) at 8 h/day, and 24.00 (95% CI, 8.93–64.50) at 9 h/day (Table 3). The estimated association rose steeply between 6 and approximately 8 h/day and subsequently showed a plateau-like pattern, with increasing uncertainty toward the upper end of the common-support range (Figure 5).

Neither age nor sex was significantly associated with headache presentation in the primary model (age: aOR = 0.98, 95% CI, 0.92–1.04, *p* = 0.450; female sex: aOR = 1.59, 95% CI, 0.66–3.82, *p* = 0.300). Previous migraine diagnosis showed a positive association that did not reach conventional statistical significance in the primary model (aOR = 3.28, 95% CI, 0.98–10.95; *p* = 0.053).

In the extended model additionally incorporating sleep duration and sleep quality, the nonlinear association between smartphone screen time and headache presentation remained statistically significant (overall association and nonlinear component, both *p* < 0.001). Relative to 6 h/day, the corresponding aORs were 10.37 (95% CI, 5.40–19.90) at 7 h/day, 29.47 (95% CI, 11.56–75.10) at 8 h/day, and 29.04 (95% CI, 9.86–85.50) at 9 h/day.

In this extended model, usual sleep duration was positively associated with headache presentation (aOR per additional hour = 2.13, 95% CI, 1.21–3.74; *p* = 0.009), whereas sleep quality was not independently associated with headache presentation after simultaneous adjustment (aOR per 1-point increase = 0.88, 95% CI, 0.71–1.09; *p* = 0.239). Previous migraine diagnosis was also associated with headache presentation in the extended model (aOR = 3.83, 95% CI, 1.07–13.70; *p* = 0.039). Age and sex remained nonsignificant (Table 3).

## 4. Discussion

In this case–control study of young adults presenting to the ED, device-recorded smartphone screen time was substantially higher among participants with headache than among ED controls and was moderately associated with greater headache severity within the headache group. The association between smartphone screen time and headache presentation was strongly nonlinear after adjustment for age, sex, and previous migraine diagnosis. Participants with headache also reported poorer sleep quality and somewhat shorter usual sleep duration; however, the relationships among screen time, sleep characteristics, and headache were more complex after multivariable adjustment.

Previous studies have linked greater screen exposure with headache frequency and severity, particularly among young adults and individuals with migraine [7,16,17,18]. Our findings extend this evidence to a clinically selected ED population. The positive correlation between smartphone screen time and headache severity provides complementary within-group evidence supporting an association between greater smartphone exposure and headache burden. Several mechanisms may contribute to this relationship, including visual strain, prolonged near work, behavioral patterns associated with extended device use, and sleep disruption [2,7]. Nevertheless, the present design cannot establish directionality. Greater screen exposure may contribute to headache symptoms, but individuals prone to headache may also exhibit different patterns of smartphone use.

The association between smartphone screen time and headache presentation was nonlinear, with a steep increase in adjusted odds between 6 and approximately 8 h/day followed by relative flattening within the common-support range. This pattern suggests that the relationship cannot be adequately summarized by a constant effect per additional hour of screen exposure. Nevertheless, the magnitude of the estimated associations warrants caution. Because these estimates were derived from a case–control sample with limited overlap in screen-time distributions at higher exposure levels, they should not be interpreted as population risks or causal effects. Moreover, the shape of the association does not establish a clinically meaningful threshold or a causal dose–response relationship.

Participants with headache also reported poorer sleep quality and shorter usual sleep duration than controls, consistent with previous evidence linking sleep disturbances with headache disorders [1,9,19]. However, these between-group differences were not accompanied by clear within-group associations with headache severity. Neither sleep duration nor sleep quality was significantly correlated with headache severity among participants with headache, and sleep quality was not independently associated with headache presentation after multivariable adjustment. These findings suggest that sleep disturbances may accompany headache presentation without necessarily exhibiting a simple relationship with headache severity in this population.

The association between sleep duration and headache presentation was less straightforward. Although participants with headache reported shorter sleep duration in the unadjusted comparison, longer sleep duration was positively associated with headache presentation in the extended multivariable model. Given the complex and potentially bidirectional relationships among sleep, smartphone exposure, and headache, this finding should be interpreted cautiously. The adjusted association should not be interpreted as evidence that longer sleep increases the risk of headache; rather, its change in direction indicates that the relationship between sleep duration and headache was sensitive to multivariable adjustment and may also reflect residual or unmeasured confounding.

Previous studies across different populations have consistently associated greater or evening smartphone use with poorer sleep outcomes [20,21,22]. The relationship between smartphone screen time and sleep quality was also weaker than suggested by the pooled analysis alone. Although greater screen time was associated with poorer sleep quality when all participants were considered together, the association was attenuated after accounting for study-group membership, and within-group correlations were modest. Moreover, there was no evidence that the strength of this association differed between the headache and control groups. Thus, part of the pooled correlation likely reflects between-group differences in both smartphone screen time and sleep quality rather than a strong within-group association.

An important methodological feature of this study was the use of device-recorded smartphone screen time rather than participant-recalled exposure. Screen time was derived from the most recently completed seven-day period and excluded the day of ED presentation, thereby reducing reliance on recall and the potential influence of acute ED attendance on the exposure estimate. Nevertheless, native smartphone tracking is not equivalent to a fully standardized measure of total digital exposure. Differences in operating systems and device settings, use of multiple smartphones, and exposure to computers, tablets, and other screens were not captured. The findings should therefore be interpreted specifically in relation to recorded smartphone screen time rather than overall screen exposure.

These findings may have clinical relevance because smartphone exposure and sleep characteristics are readily assessable contextual factors in young adults presenting with headache. However, the present study does not establish a clinically actionable screen-time threshold, its predictive value, or the benefit of reducing smartphone use. Accordingly, smartphone screen time should not yet be regarded as a screening or treatment target based on these data alone. Prospective longitudinal studies incorporating standardized headache diagnoses, more comprehensive assessments of digital exposure and sleep, and repeated headache measurements are needed to clarify temporality and determine whether modifying these factors influences headache burden.

## 5. Limitations

This study has several limitations. First, the case–control design does not allow the temporal sequence of the observed associations among smartphone screen time, sleep characteristics, and headache to be established; therefore, causality cannot be inferred, and reverse causation remains possible. Second, the use of hospital-based ED controls may have introduced selection bias, including the possibility of Berkson’s bias. Detailed diagnostic and presenting-complaint categories of the control participants were not recorded, preventing assessment of whether any particular diagnosis or diagnostic category predominated. In addition, acute illness, pain, or anxiety among ED controls may have independently influenced sleep characteristics or smartphone-use patterns. Third, headache subtypes were not prospectively classified according to standardized diagnostic criteria such as the International Classification of Headache Disorders, resulting in a clinically heterogeneous headache group and preventing assessment of whether the observed associations differed among migraine, tension-type headache, or other headache phenotypes. Undiagnosed headache disorders and other unmeasured factors may therefore have contributed to residual confounding. Fourth, several variables, including usual sleep duration, migraine history, and headache characteristics, were self-reported, and sleep duration was not objectively verified. Although validated instruments were used, headache severity and sleep quality were assessed using single-item measures (NRS and SQS, respectively), which may provide a less comprehensive characterization of these constructs than multidimensional instruments. Fifth, although smartphone screen time was obtained from device-recorded data rather than participant recall, native tracking systems are not fully standardized across devices and operating systems. Multiple-smartphone use, application-specific exposure, occupational versus recreational use, and exposure to computers, tablets, televisions, and other digital devices were not assessed; thus, the exposure measure represents recorded smartphone screen time rather than total digital exposure. Sixth, potentially important confounders, including medication use, caffeine intake, stress, anxiety, depression, other psychiatric conditions, and additional medical or behavioral factors, were not systematically measured and therefore could not be included in the adjusted analyses. Finally, the single-center setting, restriction to adults aged 18–40 years, and limited overlap in screen-time distributions between the headache and control groups, particularly at higher exposure levels, may limit generalizability and increase uncertainty regarding the magnitude of the adjusted odds ratios. These limitations should be considered when interpreting the findings, which should be regarded as hypothesis-generating and require external validation in larger, prospectively characterized populations.

## 6. Conclusions

In this case–control study of young adults presenting to the ED, longer device-recorded smartphone screen time was associated with headache presentation and greater headache severity. The association between smartphone screen time and headache presentation was nonlinear, although the magnitude of the adjusted estimates should be interpreted cautiously. Participants with headache also reported poorer sleep quality and shorter usual sleep duration than controls, but these sleep characteristics showed more complex relationships with headache after multivariable adjustment. These findings identify smartphone screen time and sleep characteristics as relevant contextual factors associated with headache presentation in young adults but do not establish causality, a clinically actionable screen-time threshold, or the benefit of reducing smartphone use. Larger prospective studies with standardized headache classification and more comprehensive assessment of digital exposure and sleep are needed to clarify these relationships and their potential clinical implications.

## Figures and Tables

**Table 1:** Demographic and clinical characteristics of the study participants.

Variable	Headache	Control	*p* Value	Effect_Size
Age, years	27.0 (21.0–33.0)	27.5 (22.0–34.0)	0.366	*r_rb_* = −0.060
Female sex	92 (61.3%)	80 (53.3%)	0.161	*V* = 0.081
Previous migraine diagnosis	34 (22.7%)	28 (18.7%)	0.392	*V* = 0.049
Smartphone screen time, hours/day	7.0 (7.0–8.0)	5.0 (4.0–5.0)	<0.001	*r_rb_* = 0.857
Sleep duration, hours/day	7.0 (7.0–8.0)	8.0 (7.0–8.0)	0.005	*r_rb_* = −0.175
Sleep quality score	5.0 (4.0–6.0)	7.0 (6.0–8.0)	<0.001	*r_rb_* = −0.387

Continuous variables are presented as median (interquartile range [IQR]) and were compared using the Mann–Whitney *U* test. Categorical variables are presented as *n* (%) and were compared using Pearson’s chi-square test. Rank-biserial correlation (*r_rb_*) and Cramer’s *V* (*V*) are reported as effect-size measures for continuous and categorical variables, respectively.

**Table 2:** Self-reported headache characteristics of participants in the headache group.

Characteristic	Headache Group (*n* = 150)
Self-reported headache frequency	
Less than once per week	132 (88.0)
Once per week	11 (7.3)
More than once per week	7 (4.7)
Headache severity, NRS, median (IQR)	6.0 (4.0–7.0)

NRS, Numeric Rating Scale; IQR, interquartile range. Headache frequency was self-reported using predefined categorical response options.

**Table 3:** Adjusted associations between smartphone screen time and headache presentation using nonlinear logistic regression models.

Variable	Primary Model, aOR (95% CI)	*p*-Value	Extended Model, aOR (95% CI)	*p*-Value
Smartphone screen time		<0.001		<0.001
6 h/day	Reference		Reference	
7 h/day	8.83 (5.00–15.60)		10.37 (5.40–19.90)	
8 h/day	23.70 (10.38–54.10)		29.47 (11.56–75.10)	
9 h/day	24.00 (8.93–64.50)		29.04 (9.86–85.50)	
Nonlinear component		<0.001		<0.001
Age, per year	0.98 (0.92–1.04)	0.450	0.98 (0.92–1.04)	0.543
Female sex	1.59 (0.66–3.82)	0.300	1.63 (0.66–4.03)	0.286
Previous migraine diagnosis	3.28 (0.98–10.95)	0.053	3.83 (1.07–13.70)	0.039
Sleep duration, per h/day			2.13 (1.21–3.74)	0.009
Sleep quality, per 1-point increase			0.88 (0.71–1.09)	0.239

aOR, adjusted odds ratio; CI, confidence interval. Smartphone screen time was modeled using a natural cubic spline with 2 degrees of freedom. Odds ratios for smartphone screen time are presented relative to 6 h/day. The primary model was adjusted for age, sex, and previous migraine diagnosis. The extended model additionally included sleep duration and sleep quality. Screen-time odds ratios are displayed for 6–9 h/day because this was the observed range in which both headache cases and controls were represented. The overall association of smartphone screen time and the nonlinear component were statistically significant in both models (*p* < 0.001).

## Data Availability

The data supporting the findings of this study are available from the corresponding author upon reasonable request. The study includes data collected from human participants. Public sharing of the dataset is restricted to protect participant privacy and to comply with the conditions of the Institutional Review Board approval. De-identified data supporting the findings of this study are available from the corresponding author upon reasonable request, provided that the request complies with applicable ethical and institutional requirements.

## Supplementary Materials

The following supporting information can be downloaded at: https://www.mdpi.com/article/10.3390/healthcare14162657/s1, File S1: Data Collection Form.

## Abbreviations

NRS	Numeric Rating Scale
SQS	Sleep Quality Scale
PSQI	Pittsburgh Sleep Quality Index
MQI	Morning Questionnaire–Insomnia
IQR	Interquartile Range
OR	Odds Ratios
CI	Confidence Interval
VIFs	Variance Inflation Factors
ED	Emergency Department
