# Supplementary material for: Smartphone Screen Time, Sleep Quality, and Headache in Young Adults Presenting to the Emergency Department: A Case–Control Study
Source: Healthcare (Basel). 2026 Aug 21;14(16):2657. doi: 10.3390/healthcare14162657 (PMC13512605; doi:10.3390/healthcare14162657)
Supplement: Supplementary file 1 [file healthcare-14-02657-s001.zip › healthcare-4494502-supplementary.pdf]

Smartphone Screen Time, Sleep Quality, and Headache in Young Adults Presenting to the Emergency Department: A Case–Control Study

File S1. Data Collection Form

1. Age	_____ years
2. Sex	☐ Male ☐ Female
3. Previous migraine diagnosis	☐ Present ☐ Absent
4. Presenting complaint	☐ Headache ☐ Other

5. Headache frequency (for participants presenting with headache)

☐ Less than once per week

☐ Once per week

☐ More than once per week

6. Headache severity (for participants presenting with headache)

Recorded using the 11-point Numeric Rating Scale (NRS), from 0 (no pain) to 10 (worst imaginable pain), as described in the Methods and cited reference of the main manuscript.

7. Smartphone screen time (all participants)

Average daily smartphone screen time: _____ h/day. The value was recorded from the smartphone's built-in screen-time report using the preceding completed 7-day period, excluding the day of ED presentation, according to the procedure described in the Methods.

8. Usual sleep duration (all participants)

Usual sleep duration: _____ h/day.

9. Sleep quality (all participants)

Assessed using the validated Turkish version of the Single-Item Sleep Quality Scale (SQS) for the previous 7 days. The instrument is not reproduced here; administration, scoring, psychometric properties, and the relevant references are provided in the Methods of the main manuscript.

Note: This supplementary file presents the structure of the study-specific data collection form in a publication-ready format. It does not reproduce third-party scale graphics or full copyrighted instrument content.
